# Adhesion Evaluation of an Embedded SiN/GaAs Interface Using a Novel “Push-Out” Technique

**DOI:** 10.3390/mi14010037

**Published:** 2022-12-23

**Authors:** Sahar Dehkhoda, Mingyuan Lu, Han Huang

**Affiliations:** School of Mechanical and Mining Engineering, The University of Queensland, Brisbane, QLD 4072, Australia

**Keywords:** adhesion, embedded interface, multilayer, delamination

## Abstract

Adhesion assessments of an embedded interface in a multilayer system that contains a ductile layer are challenging. The occurrence of plastic deformation in the ductile layer often leads to additional complexity in analysis. In this study, an innovative “push-out” technique was devised to evaluate the interfacial toughness (*G_in_*) of the embedded SiN/GaAs interface in a Au/SiN/GaAs multilayer system. Focus ion beam (FIB) milling was utilized to manufacture the miniaturized specimen and scratching with a conical indenter was used to apply load. This approach effectively minimized plastic deformation in the soft Au layer while inducing tensile stress to the embedded SiN/GaAs interface. As a result, the Au/SiN bilayer detached from the GaAs substrate with little plasticity. The energy associated with the interfacial delamination was derived from analyzing the load–displacement curves obtained from the scratching test. The *G_in_* of the SiN/GaAs interface was calculated by means of energy analysis, and the average *G_in_* was 4.86 ± 0.96 J m^−2^.

## 1. Introduction

Interfacial failure is a significant issue in microelectronic devices which comprise complex arrays of multilayer film structures [1,2,3]. The lack of reliable techniques for evaluating the properties of a variety of interfaces in integrated circuitries continues to pose a significant challenge in the design of new devices and manufacturing process improvement [4]. In particular, techniques for assessing the adhesion of embedded interfaces in a multilayer structure are highly sought-after [5,6,7]. 

Various micro-mechanical testing techniques have been developed to characterize interfacial adhesion. Nanoindentation [8,9,10,11,12], nanoscratch [13,14], and micro-cantilever (MC) and micro-bridge (MB) bending [7,15,16,17,18] are widely used. From these, nanoscratch is considered to be a semi-quantitative approach [19], whereas top-surface nanoindentation has been used to measure the interfacial strength and toughness of a stiff dielectric film attached onto a comparatively ductile semiconductor substrate [19,20]. MC and MB bending tests have been used to evaluate interfacial toughness, both applicable to embedded interfaces [7,15,17]. However, the suitability of MC and MB bending is limited to the interface of a stiff film on an ideally stiff substrate [18], as bending induced plastic deformation in ductile layers could prevent interfacial delamination from occurring or introduce additional complexity and substantial error to the analysis [21]. Hence, an interfacial adhesion quantification technique that can cope with plasticity is badly needed. 

Metal thin film interconnects and electrodes are common elements in integrated circuits. An archetypical structure is a Au film patterned on a SiN passivated GaAs substrate [18,22]. Failure in the embedded SiN/GaAs interface can result in the loss of gate control in capacitors and moisture-incursion-induced substrate degradation [23,24], impairing the performance and service life of devices. Thus, adhesion evaluations of these ceramic/semiconductor interfaces are of primary importance for improving the performance and reliability of the devices. The challenge of quantitative property assessment of the embedded SiN/GaAs interface, where extensive plastic deformation of the Au film occurs readily, is yet to be resolved. Hence, an alternative testing approach is needed, which is capable of introducing stresses to the embedded SiN/GaAs interface, while minimizing plastic deformation in the ductile Au layer.

In this study, a new push-out test was designed to evaluate the interfacial properties of a SiN/GaAs interface embedded in a Au/SiN/GaAs multilayer structure. The miniaturized specimens were fabricated using focused ion beam (FIB) milling and a conical indenter was utilized to apply lateral loading via scratching, to detach the Au/SiN bilayer structure from the GaAs substrate. This approach can significantly reduce plasticity in the Au layer, allowing for an accurate energy-based analysis to quantify the interfacial toughness.

## 2. Methodology

### 2.1. Sample Preparation

**Figure 1:**
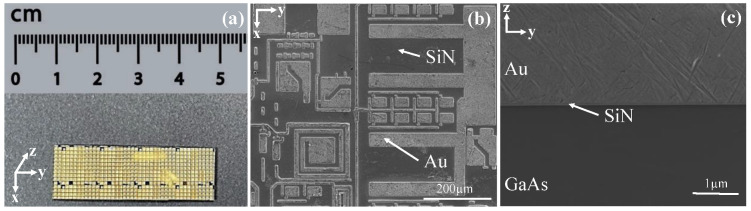
(**a**) A photograph of the Au patterned SiN/GaAs sample; SEM micrographs of (**b**) top and (**c**) cross-sectional surfaces of the Au/SiN/GaAs multilayer sample.

The SiN passivated GaAs wafer with Au patterns was supplied by WIN semiconductors Co, as shown in Figure 1a. The wafer was cut into small rectangular coupons, mounted into epoxy resin, ground, and polished to attain a flat and smooth cross-sectional surface. The resin block was dissolved in dichloromethane after polishing and the sample was collected, rinsed using distilled water and dried using compressed air. The polished cross-sectional samples were examined using a 7100F scanning electron microscope (SEM, JEOL Ltd., Tokyo, Japan). The SEM image of a typical cross-section is shown in Figure 1c. The thickness of the SiN film and the Au top layer were 0.08 µm and 3.32 µm, respectively. The elastic modulus (*E*) and hardness (*H*) of the Au layer, SiN film and GaAs substrate were measured using nanoindentation (TI900 Triboindenter, Hysitron Inc., Eden Prairie, MN) [25]. A Berkovich indenter with an included angle of 142.3° and a tip radius of 100 nm was used for indenting. The *E* and *H* of the three components are given in Table 1. 

### 2.2. Focused Ion Beam (FIB) Milling of Push-Out Specimens

**Figure 2:**
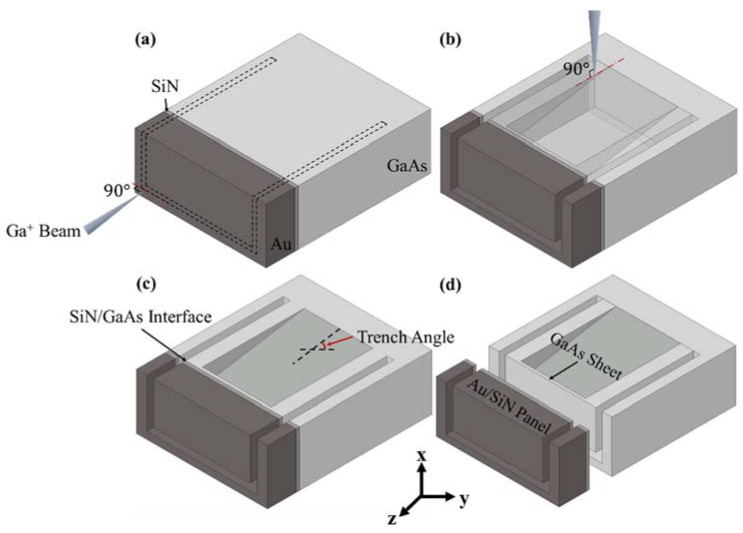
Illustration of the push-out sample milling process: (**a**) milling of the Au/SiN/GaAs rectangular prism, (**b**) milling of the trench, (**c**) final geometry of the push-out specimen, and (**d**) the GaAs thin sheet.

**Figure 3:**
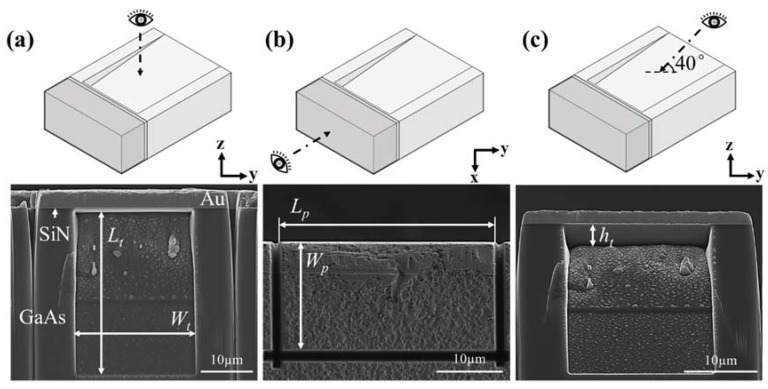
SEM micrographs of a typical FIB-milled push-out specimen (specimen S2, see Table 2 for details): (**a**) cross-sectional view; (**b**) top surface showing the Au panel; and (**c**) side view with 40° tilting angle showing the trench.

Miniaturized specimens for the push-out tests were produced using a FIB (FEI Scios Dual beam, Oregon, USA) milling technique. The milling process is illustrated in Figure 2. First, a rectangular volume was milled out by applying the ion beam perpendicular to the top surface and close to the polished edge of the coupon, as shown in Figure 2a. A milling current of 5 nA was used in this step. A rectangular Au/SiN bi-layer panel attached to the GaAs base was created. A trench was then milled into the GaAs substrate by positioning the ion beam at a right angle to the polished cross-section, as shown in Figure 2b. The milling current used was 5 nA. As shown in Figure 2c,d, the inner face at the deeper end of the trench was parallel to the SiN/GaAs interface and a very thin sheet of GaAs was left attached to the SiN film. The thickness of the GaAs sheet is in the range of 60–80 nm. Five specimens in total were fabricated. SEM images of a typical push-out specimen are shown in Figure 3. The depth (*h*_t_), length (*L*_t_), and width (*W*_t_) of the trench, as well as the length (*L*_p_) and width (*W*_p_) of the rectangular Au/SiN panel, were measured using SEM, as given in Table 2. 

### 2.3. Push-Out Test

**Figure 4:**
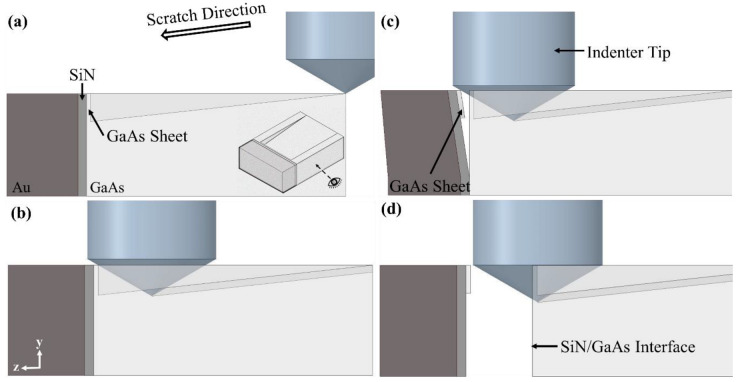
Schematic illustration of the push-out test: (**a**) indentation with normal load of 2 mN just outside the edge of the trench; (**b**) scratching along the central line of the trench until the indenter contacted the Au/SiN/GaAs panel; (**c**) fracture of the GaAs thin sheet and peeling of the Au/SiN panel off the GaAs base; and (**d**) complete detachment of the Au/SiN panel from the GaAs base.

Scratching was performed on the Triboindenter to apply a lateral load to the specimen to induce failure in the SiN/GaAs interface. A conical indenter, with a spherical apex, was used for scratching, and had an included angle of 120° and a tip radius of 5 µm. The sample was mounted with the polished cross-section perpendicular to the indenter. Alignment of both the indenter and the specimen was performed prior to testing to ensure the sample surface was flat and the scratch direction was along the central line of the trench. The configuration of the push-out test is illustrated in Figure 4. As shown, the indenter first approached the sample from outside the edge of the trench and a normal load of 2 mN was applied (Figure 4b). The indenter then moved along the intended scratch direction with a translational velocity of 0.5 µm/s toward the Au/SiN/GaAs panel, while the normal load remained constant. The indenter then contacted the GaAs sheet attached onto the Au/SiN bilayer, as shown in Figure 4c. The GaAs sheet subsequently fractured close to its edge. As the indenter continued pushing the panel, tensile stress was generated at the SiN/GaAs interface. The panel was thus peeled off from the GaAs substrate, as illustrated in Figure 4d, eventually leading to the detachment of the Au/SiN panel, as shown in Figure 4e. The lateral load–displacement (*P–h*) data from the scratch test were collected and analysed. 

## 3. Results and Discussion

### 3.1. Interfacial Delamination

**Figure 5:**
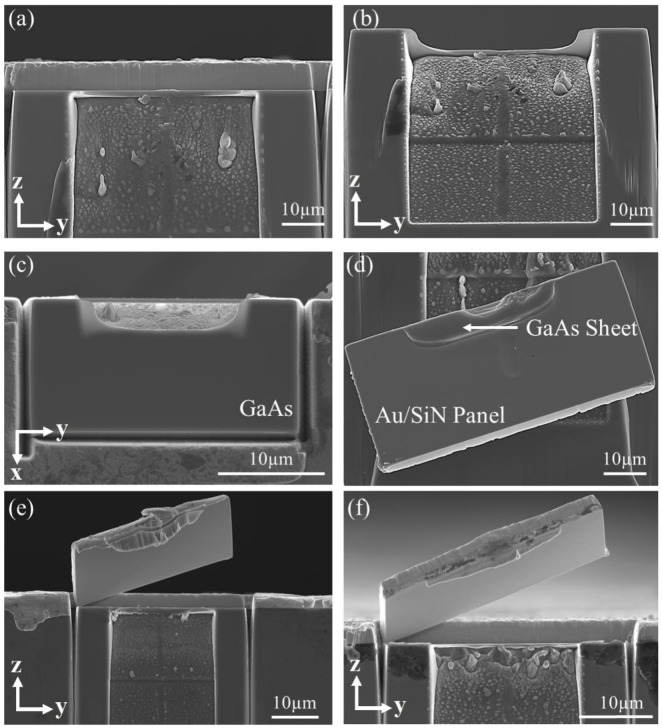
SEM post-mortem examination of a typical specimen (S2) after the push-out test, showing the detachment of the SiN/Au panel from the GaAs substrate: (**a**) cross-section; (**b**) side view with 40° tilting angle; (**c**) top surface showing the fresh exposed GaAs surface after the Au/SiN panel was peeled off; (**d**) the detached SiN/Au panel (the panel was picked up by a micro-manipulator and placed on the sample for SEM observation; the GaAs thin sheet separated from the substrate can be seen); the detached panels of S1 and S5 are provided in (**e**,**f**), respectively.

SEM images of a specimen (S2) after the push-out test are shown in Figure 5. As indicated, the Au/SiN panel detached entirely from the GaAs substrate. The exposed GaAs and SiN surfaces are shown in Figure 5c,d, respectively, which are smooth and clean. This indicated that the crack only propagated within the SiN/GaAs interface and across the entire area. Notably, the detached Au/SiN panel was picked up with a micro-manipulator using a tungsten needle and placed on the sample for SEM observation (see Figure 5d). The SEM images of S1 and S5 are also given in Figure 5e,f, respectively, to show the integrity of the detached panels from different angels. Apart from the localized deformation in the GaAs sheet, no distortion or plastic deformation were observed for the Au/SiN panels. It was also observed that the GaAs sheet was damaged locally due to the concentrated contact stress applied by the moving indenter. The presence of the GaAs panel significantly reduced the pressure applied locally to the Au/SiN panel. Plastic deformation of Au layer was minimized as a result.

**Figure 6:**
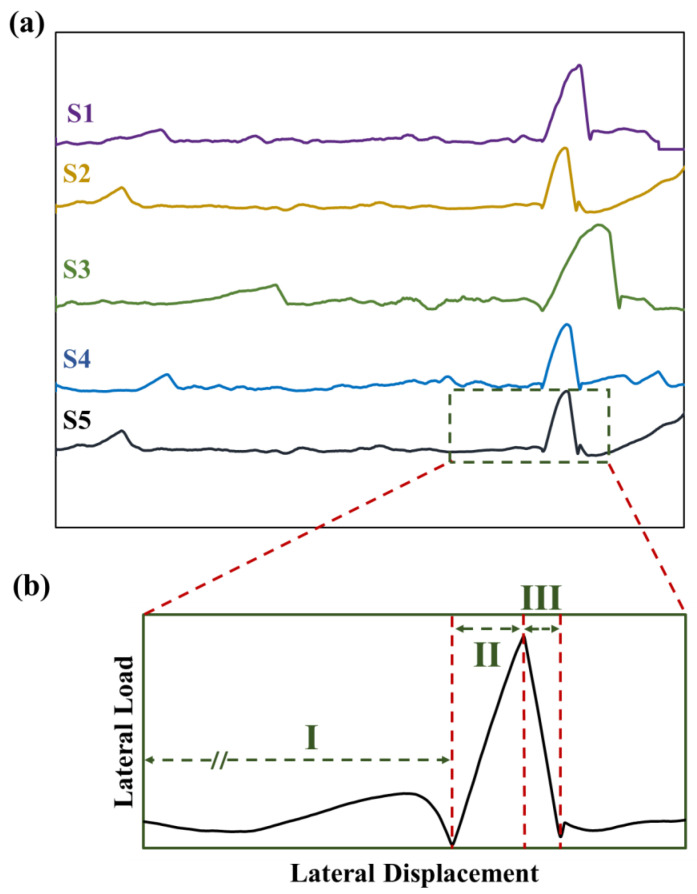
(**a**) P–h curves obtained from the push-out tests and (**b**) the three distinct stages (Stage I, II and III).

The *P–h* curves of the push-out tests are provided in Figure 6a. Three distinct stages can be identified, shown in Figure 6b. In Stage I, the indenter translated along the central line of the trench with a contact load of 2 mN in the normal direction (Figure 4b,c). The minor peaks on the *P–h* curves in this regime were associated with the indenter tip moving over small protrusions at the bottom of the trench. These protrusions are formed as a result of material redeposition during the ion sputtering process from milling the sample [26,27]. Toward the end of the trench, the indenter contacted the Au/SiN/GaAs multilayer, as demonstrated in Figure 4b. In Stage II, the lateral load increased sharply as the Au/SiN/GaAs multilayer structure resisted deflection, obstructing the indenter from moving forward. At the end of stage II, the GaAs thin sheet fractured close to the edge where the bending moment was the highest and was then separated from the substrate (Figure 4c). In Stage III, the tip continued to move forward, pushing the Au/SiN/GaAs multilayer. The resultant lateral force led to the peeling of the Au/SiN panel off the GaAs base until it was completely detached from the substrate along the interface (Figure 4d); this resulted in a rapid drop of lateral load due to the advancing of the interfacial crack. 

### 3.2. Interfacial Toughness (G_in_)

**Figure 7:**
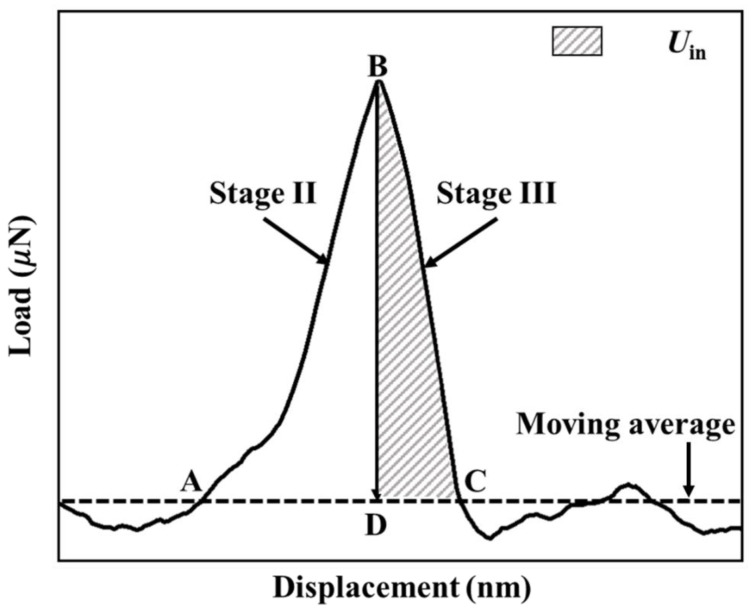
The P–h curve of a push-out test, where the shaded area (DBC, Stage III) gives the energy U_in_ associated with the delamination event, while area ABD (Stage II) gives the energy used in deflecting the Au/SiN/GaAs multilayer panel.

Energy analysis was carried out to calculate the interfacial toughness, *G_in_*, of the SiN/GaAs interface. The total energy associated with the detachment of the Au/SiN panel from the GaAs substrate, *U_in_*, is the area DBC underneath the load–displacement curve in Stage III shown in Figure 7. As mentioned earlier, the fracture surface shown in Figure 5c,d is smooth, indicating that interfacial crack did not deflect into the Au/SiN panel or GaAs substrate. Hence, the area of interfacial fracture, *A_in_*, can be accurately measured using the top-surface SEM image shown in Figure 5c. On the other hand, the plastic deformation that has occurred during the scratching generates energy, which by introducing lateral load baseline (through the moving average), it is subtracted from total detachment energy. The toughness of the SiN/GaAs interface can then be calculated using [28,29,30],

(1) $$G_{in}=U_{in}/A_{in}$$

**Figure 8:**
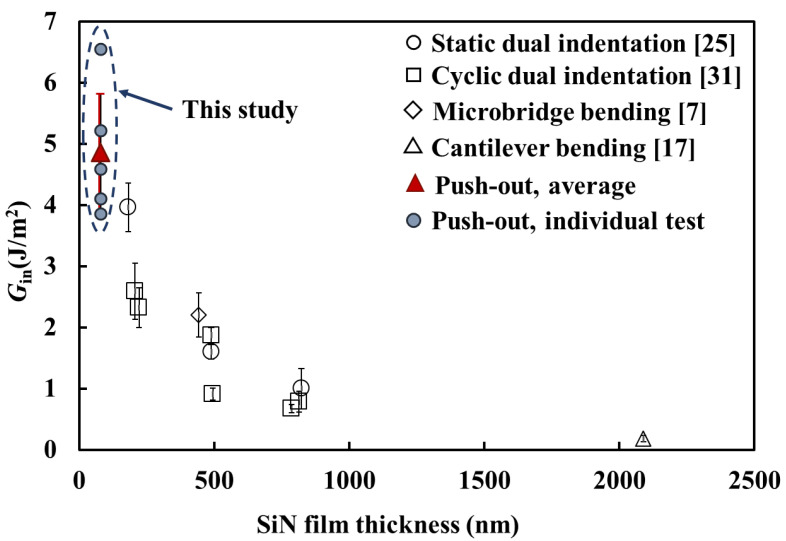
The G_in_ values of a variety of SiN/GaAs interface measured using different micro-mechanical testing techniques, plotted against the thickness of SiN film.

The values of *U_in_*, *A_in_* and *G_in_* of the SiN/GaAs interface are given in Table 3. The mean *G_in_* is 4.86 ± 0.96 J m^−2^. In Figure 8, the *G_in_* value obtained from this study is compared with those measured using different techniques in previous studies [7,17,25,31]. As shown, the values from the previous work are in the ranges from 3.96 ± 0.40 – 0.18 ± 0.05 J m^−2^ while the thickness of the SiN film is in the range of 182-2090 nm. It was found that *G_in_* decreased with the increase in SiN film thickness; this was likely attributed to the increase in thermal stresses in the SiN film and change in the structure of the interfacial region, which weakened the interface [31]. The complex effect of thickness is not in the scope of this work and will be studied in the future. The mean *G_in_* value of the embedded SiN film in this study is 4.86 ± 0.96 J m^−2^, which is the highest (see Figure 8). The thickness of the SiN film is approximately 80 nm, which is the lowest. Therefore, the high *G_in_* was expected, which agrees well with our previous study. 

**Figure 9:**
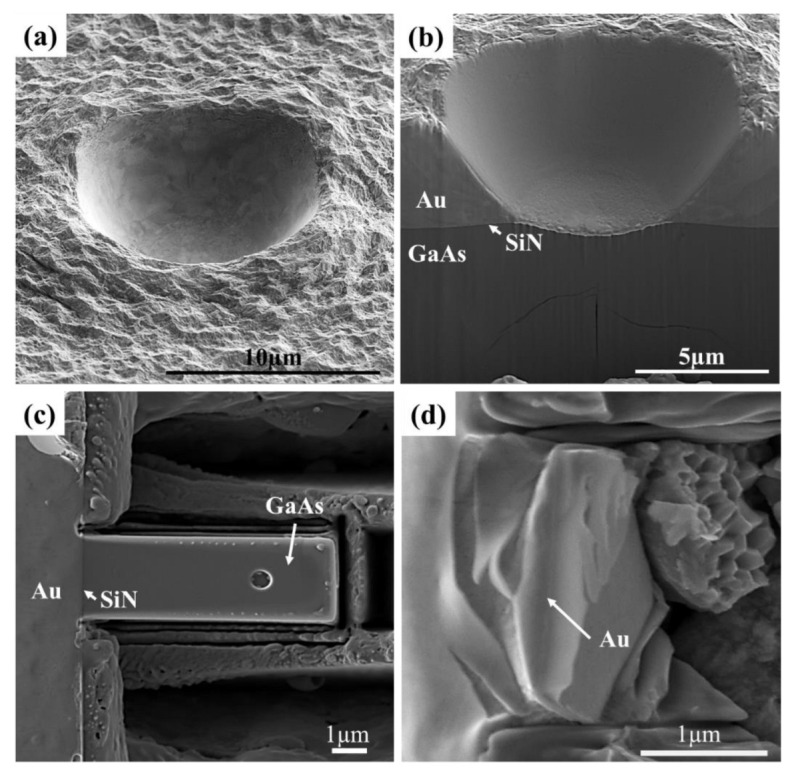
SEM micrographs of (**a**) an impression from a top-surface indentation showing appreciable plastic deformation in Au, (**b**) cross-section of the impression, (**c**) a micro-cantilever for the bending test, (**d**) post-mortem examination showing the detachment of GaAs cantilever from the base and the significant plastic deformation in Au.

Top-surface indentation and cantilever bending were used to test the Au/SiN/GaAs multilayer system, which were successfully applied to measure the adhesion of SiN/GaAs interface in the previous studies [17,25]. Top-surface indentation induced pronounced plastic deformation in the Au layer, leading to a marked indent impression without interfacial delamination observed, as shown in Figure 9a,b. Micro-cantilever bending resulted in the detachment of GaAs from SiN/Au layer. However, extensive plastic deformation occurred in the Au layer before interfacial delamination commenced, as shown in Figure 9c,d. The occurrence of plastic deformation of Au was a major obstacle for quantitatively assessing the adhesion of the embedded SiN/GaAs interface as it introduced significant complexity to the analysis.

The push-out test developed in this study can effectively evaluate the interfacial toughness of an embedded SiN/GaAs interface in a Au/SiN/GaAs multilayer system. It complements the top-surface indentation [25,31], micro-cantilever (MC) [17] and micro-bridge (MB) bending [7] techniques. Moreover, the FIB milling process for preparing a specimen took less than 15 min and could be automated, making this approach more efficient than MC and MB bending. Nevertheless, a potential error may be introduced, as the localized contact deformation in the GaAs thin sheet, as shown Figure 5d, consumed energy. However, this deformation was believed to occur at the end of Stage II, where the contact stress was the highest. Thus, the energy was not added to *U_in_*. In addition, the fracture of GaAs thin sheet could introduce error to the calculation because GaAs is a very brittle material, and the sheet is also very thin, typically in the range of 60–80 nm. Thus, the energy associated with the fracture of this thin brittle sheet was likely very low. This derivation was supported by the evidence that no discontinuity in the P–h curve was observed at the peak between Stages II and III, as shown in the insert of Figure 6. Hence, the energy relevant to fracture of the GaAs sheet was negligible. 

Overall, the push-out technique is capable of examining an embedded interface in a complex multilayer system and minimizing plasticity in the ductile layer, which is critical for interfacial toughness quantification. Given the simplicity of the testing configuration, this technique could also be applied to a large variety of multilayer systems containing ductile layers of different materials. Nonetheless, the applicability of this approach is limited by the resolution (accuracy) of the FIB milling process. In addition, excessive deflection of a thin ductile layer may still cause undesirable plastic deformation. Hence, this method is not suitable for multilayers with a total thickness of only a few hundred nanometres or less. However, the range of thickness that this method can apply to could not be one-size-fits-all, as it is dependent on the mechanical properties of each layer.

## 4. Conclusions

A push-out technique based upon scratching was developed for evaluating the toughness of an embedded SiN/GaAs interface in a Au/SiN/GaAs multilayer system. This approach can effectively induce tensile stress at the SiN/GaAs interface to generate delamination, while causing little plasticity in the top layer of Au. The interfacial toughness, *G_in_*, of the interface was computed by energy analysis of the load–displacement data. The mean *G_in_* of the SiN/GaAs interface in this study was 4.86 ± 0.96 J m^−2^, which was higher than those reported in previous studies. The difference in SiN film thickness was attributed to the discrepancy. 

## Figures and Tables

**Table 1:** Thickness and mechanical properties of GaAs, SiN, and Au.

Material	Thickness (µm)	Elastic Modulus (GPa)	Hardness (GPa)
Au	3.3	48	0.7 ± 0.1
SiN	0.08	140	12.7
GaAs	650	122	8.2

**Table 2:** Dimensions of the push-out specimens.

Specimens	*L_p_* (µm)	*W_p_* (µm)	*h_t_* (µm)	*L_t_* (µm)	*W_t_* (µm)
S1	36.0	20.0	3.8	33.5	22.5
S2	32.9	16.2	3.1	28.5	20.5
S3	32.9	16.5	2.9	28.8	20.7
S4	32.2	17.0	3.3	28.5	20.6
S5	32.6	16.0	3.8	28.5	20.6

**Table 3:** Values of energy release from interfacial delamination (U_in_), delamination area (A_in_), and toughness (G_in_) of the SiN/GaAs interface.

Specimen	*U_in_* (J)	*A_in_* (µm^2^)	*G_in_* (J/m^2^)
S1	2323.00	601.09	3.86
S2	1680.78	409.00	4.10
S3	3106.90	474.00	6.55
S4	2440.83	462.30	5.22
S5	1989.60	433.30	4.59

## Data Availability

Data will be provided upon request.
